# Supplementary material for: Rapid Molecular Diagnosis of Genetically Inherited Neuromuscular Disorders Using Next-Generation Sequencing Technologies
Source: J Clin Med. 2022 May 12;11(10):2750. doi: 10.3390/jcm11102750 (PMC9143479; doi:10.3390/jcm11102750)
Supplement: Supplementary file 1 [file jcm-11-02750-s001.zip › jcm-1640664-supplementary.pdf]

Supplementary Table S1. Clinical data and genetic findings for each patient for which a positive diagnosis was established using the INMD panel. Recurrent variants identified in our cohort are highlighted in bold.

	Patient ID	Age (years)	Sex	HPO	Variants	GnomAD	Family study	Phenomizer p-value	Gene	Disorder MIM	Inheritance
Muscular dystrophies / Myopathies	1	56	F	Limb-girdle muscular dystrophy<br>Myopathy<br>Distal/proximal muscle weakness	c.580A>T	-	*De novo*	0.0080	*ACTA1*	Nemaline myopathy<br># 161800	AD /AR
	2	8	F	Nemaline bodies<br>Muscle weakness, proximal and distal<br>Muscle weakness, upper and lower limbs	c.529A>G/<br>c.809-2A>T	-/<br>0.0000041	Carriers	0.0006			
	3	43	M	Elevated serum creatine kinase<br>Muscle weakness<br>Muscular hypotonia	c.133G>T/<br>c.242C>T/<br>c.1029A>T	-/-/-	Carriers	0.2616	*AMPD1*	Myopathy due to myoadenylate deaminase deficiency<br># 615511	AR
	4	44	M	Myopathy<br>Exercise intolerance<br>Restless leg<br>Myalgia<br>Dyslipidemia	**c.191dup/<br>c.191dup**	0.0011/<br>0.0011	Carriers	0.1776	*ANO5*	Muscular dystrophy<br># 611307	AD /AR
	5	53	M	Limb-girdle muscular dystrophy	**c.191dup/<br>c.191dup**	0.0011/<br>0.0011	Carriers	0.3181			
	6	26	F	Myopathy<br>Elevated serum creatine phosphokinase<br>Distal muscle weakness<br>Proximal muscle weakness	**c.191dup/<br>c.692G>T**	0.0011/<br>0.00103	Carriers	0.0038			
	7	52	F	Myotonia<br>Elevated serum creatine phosphokinase<br>Muscle weakness	c.1119+1G>T/<br>c.1119+1G>T	0.000008/<br>0.000008	Carriers	0.2175			
	8	62	M	Myalgia<br>Elevated serum creatine phosphokinase<br>Reduced muscle fiber dysferlin	c.206_207del/<br>c.206_207del	0.000004/<br>0.000004	Carriers	0.0607			

	Patient ID	Age (years)	Sex	HPO	Variants	GnomAD	Family study	Phenomizer p-value	Gene	Disorder MIM	Inheritance
Muscular dystrophies / Myopathies	9	41	M	Muscular dystrophy<br>Elevated serum creatine phosphokinase	c.1982T>C<br>c.1982T>C	0.000007/<br>0.000007	Carriers	0.0469	*ANO5*	Muscular dystrophy<br># 611307	AR
	11	59	F	Muscular dystrophy	**c.2361_2362insTCAT**<br>c.505C>T	-/<br>0.000004	Carriers	0.6363	*CAPN3*	Muscular dystrophy, limb-girdle<br># 618129	AD /AR
	12	58	M	Limb-girdle muscular dystrophy	**c.2361_2362insTCAT**<br>**c.2361_2362insTCAT**	-/-	Carriers	0.4524			
	13	20	M	Muscular dystrophy	c.1962delC<br>c.2120A>G	-/<br>0.000151	Carriers	0.6363			
	14	31	F	Myopathy<br>Global systolic dysfunction	c.701C>T<br>c.701C>T	0.000016/<br>0.000016	Carriers	0.8865	*CHKB*	Muscular dystrophy<br># 602541	AR
	15	-	M	Muscle weakness	c.8215T>C<br>c.6995G>A	0.0000080/<br>-	Carriers	0.7080	*COL12A1*	Bethlem myopathy<br># 616471	AR
	16	3	F	Muscle weakness<br>Joint contracture<br>Lobulated muscle fibers<br>Reduced muscle collagen VI	c.868G>A	-	*De novo*	0.0358	*COL6A1*	Ullrich congenital muscular dystrophy<br>#254090<br><br>Bethlem myopathy<br># 158810	AD /AR
	17	47	M	Reduced muscle collagen VI<br>Myopathy	c.739-2A>G	-	*De novo*	0.0761			
	18	10	M	Myopathy	**c.1806C>A**	-	Paternal (affected)	0.0020	*COL6A2*	Ullrich congenital muscular dystrophy<br>#254090<br><br>Bethlem myopathy<br># 158810	AD /AR
	19	21	M	Nonprogressive muscular atrophy<br>Elevated serum creatine phosphokinase<br>Myopathy	**c.1806C>A**<br>c.2891T>C	-/<br>0.00000407	Carriers	0.0803	*COL6A2*	Ullrich congenital muscular dystrophy<br>#254090<br><br>Bethlem myopathy<br># 158810	AD /AR
	20	8	F	Limb-girdle muscular dystrophy	c.875G>A	-	*De novo*	0.0616			
	21	59	F	Limb-girdle muscle weakness	c.6320_6322del	-	Affected brothers	0.4861	*COL6A3*	Ullrich congenital muscular dystrophy<br>#254090<br><br>Bethlem myopathy<br># 158810	AD /AR

	Patient ID	Age (years)	Sex	HPO	Variants	GnomAD	Family study	Phenomizer p-value	Gene	Disorder MIM	Inheritance
**Muscular dystrophies / Myopathies**	22	42	M	Elevated serum creatine phosphokinase<br>Exercise intolerance<br>Muscular dystrophy	c.1688A>G<br>c.1688A>G	0.00216/<br>0.00216	Carriers	0.2116	*COL6A3*	Ullrich congenital muscular dystrophy<br>#254090	AD /AR
	23	39	M	Myopathy<br>Rhabdomyolysis<br>Elevated serum transaminases	c.1631G>A	0.00000398	*De novo*	0.2687		Bethlem myopathy<br># 158810	
	24	57	M	Distal muscle weakness<br>Elevated serum creatine phosphokinase	c.7525C>T	-	Maternal<br>(Unaffected)	0.1941	*DMD*	Duchenne muscular dystrophy<br># 310200	X-linked
	25	11	M	Myopathy	c.9622A>G	-	Maternal<br>(Unaffected)	0.9065			
	26	14	M	EMG: myotonic discharges<br>Muscular dystrophy	c.9563+1G>A	-	Maternal<br>(Unaffected)	0.1485			
	27	16	F	Elevated serum creatine phosphokinase<br>EMG: myopathic abnormalities	c.5697dupA	-	*De novo*	0.1986			
	28	1	F	Elevated serum creatine phosphokinase<br>Seizures<br>Hypoglycemia<br>Feeding difficulties	c.3125delA	-	*De novo*	0.6050			
	29	3	M	Elevated serum creatine phosphokinase<br>Elevated hepatic transaminases	g.(?_32430456)_(32486604_?)dup	-	Maternal<br>(Unaffected)	0.8279			
	30	9	M	Elevated serum creatine phosphokinase<br>Elevated hepatic transaminases	g.(?_32404402)_(32503241_?)del	-	Maternal<br>(Unaffected)	0.8279			
	31	71	M	Muscular dystrophy	c.5429G>A<br>c.5429G>A	-	Carriers	0.3200	*DYSF*	Miyoshi muscular dystrophy<br># 254130	AR
	32	3	F	Elevated serum creatine phosphokinase<br>Elevated aldolase level<br>Myopathy	c.545A>G<br>c.898G>A	0.000082/<br>0.0000322	Carriers	0.0525	*FKRP*	Muscular dystrophy-dystroglycanopathy<br># 606612	AR
	33	-	F	Myopathy<br>Muscle weakness<br>Increased muscle glycogen content	c.46G>C<br>c.46G>C	0.00000398/<br>0.00000398	Carriers	0.0264	*GYG1*	Glycogen storage disease XV<br>#613507	AR

	Patient ID	Age (years)	Sex	HPO	Variants	GnomAD	Family study	Phenomizer p-value	Gene	Disorder MIM	Inheritance
**Muscular dystrophies / Myopathies**	34	16	M	Dandy-Walker malformation<br>Hydrocephalus<br>Muscular dystrophy	c.149C>T<br>c.8075+1G>A	0.0000398/<br>-	Carriers	0.2056	*LAMA2*	Muscular dystrophy, limb-girdle<br># 618138	AR
	35	3	M	Myopathy<br>Cardiomyopathy	c.1606G>A	-	*De novo*	0.3835	*MYH7*	Distal myopathy<br># 160500	
	36	23	M	Abnormal muscle glycogen content<br>Muscle weakness	c.1820C>T<br>c.1820C>T	-/-	Carriers	0.2350	*PFKM*	Glycogen storage disease VII<br># 232800	AR
	37	62	F	Myalgia<br>Elevated serum creatine phosphokinase	c.2230-1G>T	-	*De novo*	0.0988	*PHKA1*	Muscle glycogenosis<br># 300559	X-linked
	38	51	M	Elevated serum creatine phosphokinase	c.784G>A	-	Maternal (unaffected)	0.6922			
	39	52	F	Progressive muscle weakness<br>Pelvic girdle muscle weakness	c.406T>C<br>c.406T>C	-/-	Carriers	0.1287	*POMT2*	Muscular dystrophy-dystroglycanopathy<br># 613158	AR
	40	21	M	Limb-girdle muscular dystrophy<br>Paranoia<br>Psychotic episodes<br>Joint pain<br>Gait disturbance<br>Scoliosis<br>Synovitis<br>Iron deficiency anemia	c.660G>A<br>c.660G>A	0.00209/<br>0.00209	Carriers	1.0000	*PYGM*	McArdle disease<br># 232600	AR
	41	15	F	Myopathy	c.10347+1G>A	0.000004	-	0.7174	*RYR1*	Central core disease<br># 117000	AD /AR
	42	1	M	Myopathy	c.4837C>T<br>c.7027G>A	0/<br>0.0000239	Carriers	0.7174			
	43	69	M	Limb-girdle muscular dystrophy	c.917C>T	0.0000159	*De novo*	0.4524			
	44	37	M	Myopathy<br>Elevated serum creatine phosphokinase	c.176C>T/<br>c.6856C>G	-/-	Maternal (affected)	0.1393			
	45	4	M	Myopathic facies<br>Unilateral ptosis<br>Muscle weakness<br>Motor delay	c.2709C>G	0.00000795	Father (unaffected)	0.0761			

	Patient ID	Age (years)	Sex	HPO	Variants	GnomAD	Family study	Phenomizer p-value	Gene	Disorder MIM	Inheritance
Muscular dystrophies / Myopathies	46	50	M	Paresthesia<br>Peripheral axonal neuropathy	c.7261G>T	0.0000318	-	0.2984	*RYR1*	Central core disease<br># 117000	AD /AR
	47	48	M	Elevated serum creatine phosphokinase<br>Rhabdomyolysis<br>Myalgia<br>Proximal muscle weakness	c.12836C>T	0.0000171	*De novo*	0.0059			
	48	6	F	Elevated serum creatine phosphokinase<br>Elevated hepatic transaminases<br>Marked muscular hypertrophy<br>Fatigue<br>Abnormal urinary color	c.724G>T<br>**c.739G>A**	0.00000398/<br>0.000112	Carriers	0.3651	*SGCA*	Muscular dystrophy, limb-girdle<br># 608099	AR
	49	10	F	Muscle weakness	**c.739G>A**<br>c.850C>T	0.000112/<br>0.000151	Carriers	0.7080			
	50	12	F	Limb-girdle muscular dystrophy<br>Elevated serum creatine phosphokinase	c.848G>A<br>c.848G>A	0.00000398/<br>0.00000398	Carriers	0.0699	*SGCG*	Muscular dystrophy, limb-girdle<br># 253700	AR
	51	2	F	Rhabdomyolysis<br>Elevated serum creatine phosphokinase	c.525delT<br>c.525delT	0.0000557/<br>0.0000557	Carriers	0.5727			
	52	18	F	Axial hypotonia<br>Muscle weakness	c.26095-1G>T<br>c.26095-1G>T	-/-	Carriers	0.4314	*SYNE1*	Emery-Dreifuss muscular dystrophy<br># 612998	AR
	53	3	M	Neutropenia<br>Short stature<br>Truncal obesity<br>Lactic acidosis<br>Proximal muscle weakness	c.527A>G	-	*De novo*	0.3639	*TAZ*	Barth syndrome<br># 302060	X-linked
	54	28	M	Progressive muscle weakness<br>Knee flexion contracture<br>Achilles tendon contracture<br>Reduced muscle collagen VI	c.255C>A<br>c.255C>A	-/-	Carriers	0.0222	*TCAP*	Muscular dystrophy, limb-girdle<br>#601954	AR

	Patient ID	Age (years)	Sex	HPO	Variants	GnomAD	Family study	Phenomizer p-value	Gene	Disorder MIM	Inheritance
**Muscular dystrophies / Myopathies**	55	51	F	Myopathy<br>Muscle Weakness<br>Myalgia<br>Skeletal muscle fatty infiltration	c.269G>A	-	Maternal (affected)	0.0217	*TPM2*	Nemaline myopathy<br># 609285	AD
	56	11	M	Myopathy	c.12411delT<br>c.105110_105111delCC	-/-	Carriers	0.6163	*TTN*	Muscular dystrophy, limb-girdle<br>#608807<br><br>Salih myopathy<br>#611705	AD /AR
	57	2	M	Arthrogryposis multiplex congenital<br>Amyoplasia<br>Severe muscular hypotonia<br>Global developmental delay	**c.38661_38665del**<br>**c.38661_38665del**	-/-	Maternal Isodisomy	0.1041			
	58	21	M	Muscular dystrophy<br>Arthrogryposis multiplex congenita	**c.38661_38665del**<br>**c.38661_38665del**	-/-	Carriers	0.1027			
	59	3	M	Myopathy	c.3034C>T<br>c.106531+1G>A	0.00000399/<br>0.00000447	Carriers	0.6163			
	60	3	F	Myopathy	c.33064C>T<br>c.102941G>A	0.00000402/<br>-	Carriers	0.6163			
	61	36	M	Limb-girdle muscular dystrophy	c.102966del<br>c.102966del	0.00000401/<br>0.00000401	Carriers	0.1648			
	62	12	F	Muscular dystrophy<br>Reduced muscle collagen VI	c.38737G>T<br>c.87019_87022del	0.0000081/<br>-	Carriers	0.0160			
	63	22	F	Myopathy	c.86992_86994delCTG insGTCTGTCAT<br>c.101608 + 1G>A	-/<br>0.00000447	Carriers	0.6163			

	Patient ID	Age (years)	Sex	HPO	Variants	GnomAD	Family study	Phenomizer p-value	Gene	Disorder MIM	Inheritance
**Peripheral Nerve Disease**	64	0,66	F	Poor head control<br>Hypotonia	c.1856C>T	-	*De novo*	0.4985	*DNM2*	Charcot-Marie-Tooth disease<br># 606482	AD /AR
	65	29	F	Progressive muscle weakness<br>Distal muscle weakness	c.628C>G	-	*De novo*	0.1225	*DYNC1H1*	Charcot-Marie-Tooth disease, axonal<br># 614228	AD
	66	19	M	Frequent falls<br>Sensorimotor polyneuropathy	c.547C>T	-	X-linked	0.2284	*GJB1*	Charcot-Marie-Tooth disease<br># 302800	X-linked
	67	15	M	Sensorimotor polyneuropathy	c.110G>C<br>c.110G>C	0.00026/<br>0.00026	Carriers	0.7919	*HINT1*	Neuromyotonia and axonal neuropathy<br># 137200	AR
	68	16	F	Acute demyelinating polyneuropathy	c.181G>A<br>c.1327C>T	-/<br>0.0000121	Carriers	0.4708	*IGHMBP2*	Charcot-Marie-Tooth disease<br># 616155	AR
	69	1	M	Joint hypermobility<br>Hypotonia	c.1580_1581insC	0.0000216	Paternal (affected)	1.0000	*INF2*	Charcot-Marie-Tooth disease<br># 614455	AD
	70	5	F	Muscular dystrophy	c.326A>G	-	*De novo*	1.0000	*MFN2*	Charcot-Marie-Tooth disease<br># 609260	AD /AR
	71	49	M	Muscular dystrophy	c.1666C<br>c.1972G>A	-/-	Carriers	-	*MME*	Charcot-Marie-Tooth disease<br># 617017	AR
	72	64	F	Congenital peripheral neuropathy	g.(?_15134208)_(15142953_?)dup	-	*De novo*	0.7919	*PMP22*	Charcot-Marie-Tooth disease<br># 118220	AD
	73	62	M	EMG abnormalities	g.(?_15164065)_(15903230_?)dup	-	-	1.0000			

	Patient ID	Age (years)	Sex	HPO	Variants	GnomAD	Family study	Phenomizer p-value	Gene	Disorder MIM	Inheritance
Peripheral Nerve Disease	74	74	F	Polyneuropathy<br>Gait disturbance<br>Dysphagia<br>Dysphonia	**c.2860C>T**<br>c.539_541del	0.000748/<br>0.0000558	Carriers	0.0326	*SH3TC2*	Charcot-Marie-Tooth Disease<br># 601596	AD /AR
	75	16	F	Demyelinating motor neuropathy	c.3325C>T<br>c.3325C>T	0.0000398/<br>0.0000398	Carriers	0.4640			
	76	26	F	Peripheral axonal neuropathy<br>Scoliosis<br>Talipes cavus equinovarus	**c.2860C>T**<br>**c.2860C>T**	0.000748/<br>0.000748	Carriers	0.8183			
	77	6	M	Sensorimotor polyneuropathy<br>Psychomotor delay	**c.2860C>T**<br>c.2640delC	0.000748/<br>-	Carriers	0.4763			
	78	49	F	Hammertoe<br>Talipes cavus equinovarus<br>Microcephaly<br>Gait imbalance<br>Deafness	c.3511C>T<br>**c.2860C>T**	0.00000796/<br>0.000748	Carriers	0.6998			
	79	51	M	Motor neuron atrophy<br>Parkinsonism<br>Frontotemporal Dementia	c.6477+4A>G<br>c.6477+4A>G	-/-	Carriers	0.0612	*SPG11*	Charcot-Marie-Tooth disease<br># 616668	AR
Neuromuscular Junction Diseases<br>Ion Channel Diseases	80	36	M	EMG: myotonic discharges	c.1261C>T	0.0000239	*De novo*	0.5448	*CLCN1*	Myotonia congenita, atypical, acetazolamide-responsive<br># 608390	AD
	81	24	M	Myotonia	c.1214C>T	-	Maternal (affected)	0.0032			
	82	15	F	Elevated serum creatine phosphokinase<br>Exercise intolerance<br>Myotonia	c.313C>T<br>c.501C>G	0.00035/<br>0.00117	Carriers	0.0047			
	83	6	M	Fatigable weakness<br>Muscle weakness<br>Respiratory insufficiency	c.583G>C<br>c.583G>C	-/-	Carriers	0.0055	*CHRNE*	Myasthenic syndrome, congenital, 4B, fast-channel<br>#616324	AR

	Patient ID	Age (years)	Sex	HPO	Variants	GnomAD	Family study	Phenomizer p-value	Gene	Disorder MIM	Inheritance
Neuromuscular Junction Diseases	84	16	F	Muscular hypotonia	c.1150C>G<br>c.574G>C	-/<br>0.00000398	Carriers	0.9697	*DPAGT1*	Myasthenic syndrome, congenital<br># 614750	AR
	85	4	M	Cold-sensitive myotonia	c.4774A>G	-	*De novo*	0.4196	*SCN4A*	Myotonia congenita, atypical, acetazolamide-responsive<br>#608390<br><br>Myasthenic syndrome<br># 614198	AD
	86	58	F	Myopathy<br>Muscle cramps<br>Muscle stiffness	c.3877G>A	0	*De novo*	0.0566			
Motor Neuron Disease	87	13	F	Muscle weakness	g.(?_34635615)_(34635855_?)del<br>g.(?_34635615)_(34635855_?)del	-/-	Carriers	0.7080	*SIGMAR1*	Spinal muscular atrophy<br># 605726	AR
	88	37	F	Muscle weakness<br>Distal amyotrophy	c.561_576del<br>g.(?_34635589)_(34635880_?)del	-/-	Carriers	0.5519			

F: female; M: male; AD: Autosomal dominant: AR: Autosomal recessive

Supplementary Table S2. Clinical data and genetic findings for patients with inconclusive diagnosis due to identification of heterozygous pathogenic / likely pathogenic variants in a recessive gene closely related to the patient's clinical phenotype, but a second variant was missing.

Patient ID	Age (years)	Sex	HPO	Variants	GnomAD	Phenomizer p-value	Gene	Disorder MIM	Inheritance
90	56	F	Muscular dystrophy<br>Elevated serum creatine phosphokinase	c.2235+2T>G	-	0.1248	*ANO5*	Muscular dystrophy<br># 611307	AR
91	20	M	Myopathy<br>Elevated serum creatine phosphokinase	**c.692G>T**<br>c.1767C>A	0.00103/<br>0.00000398	0.0469	*ANO5*	Muscular dystrophy<br># 611307	AR
92	2	M	Elevated serum creatine kinase<br>Delayed gross motor development	**c.2361_2362insTCAT**	-	0.7971	*CAPN3*	Muscular dystrophy, limb-girdle<br># 618129	AD /AR
93	52	M	Lower limb muscle weakness<br>Elevated serum creatine kinase<br>EMG: myopathic abnormalities<br>Lower limb muscle weakness<br>Muscular dystrophy<br>Pelvic girdle muscle atrophy	c.258G>C	0.00289	0.0004	*DAG1*	Muscular dystrophy-dystroglycanopathy<br># 613818	AR
94	38	M	Metabolic myopathy	c.365C>T	0.0000518	0.5961	*PGAM2*	Glycogen storage disease X<br># 261670	AR
95	37	F	Elevated serum creatine kinase<br>Cramps	c.1094C>T	0.000711	0.0040	*PYGM*	McArdle disease<br># 232600	AR
96	43	F	Muscular hypotonia<br>Joint contractures	c.264C>A	0.00156	0.5894	*RAPSN*	Myasthenic syndrome<br># 616326	AR
97	54	M	Muscle weakness<br>Spinal rigidity	c.763C>T	-	0.5712	*SEPN1*<br>*(SELENON)*	Muscular dystrophy<br># 602771	AR
98	70	M	Muscular dystrophy	c.409G>A	0.0000437	0.5575	*SGCA*	Muscular dystrophy, limb-girdle<br># 608099	AR
99	16	F	Upper limb muscle weakness<br>Ulcerative colitis<br>Hearing impairment<br>Bulbar palsy<br>Polyneuropathy<br>Scoliosis	c.595delG	-	0.1296	*SLC52A2*	Brown-Vialetto-Van Laere síndrome<br># 614707	AR

Supplementary Table S3. Clinical data and genetic findings for patients with inconclusive diagnosis.

Patient ID	Age (years)	Sex	Variants	Gene	Disorder MIM	Inheritance
**Possible cases of dual diagnosis**						
100	5	F	c.1287+5G>A<br>c.1287+5G>A	*TRAPPC11*	Muscular dystrophy, limb-girdle<br># 615356	AR
			c.1094C>T<br>c.1094C>T	*PYGM*	McArdle disease<br># 232600	AR
101	3	M	c.(3162+1_3163-1)_(4070+1_4071-1)dup	*DMD*	Duchenne muscular dystrophy<br># 310200	AD / AR
			c.409G>A	*MYH7*	Distal myopathy<br># 160500	
**VUS identification and missing family studies**						
102	57	F	c.11_15del	*AARS1*	Charcot-Marie-Tooth disease, axonal, type 2N	*AD*
103	50	M	c.986G>A	*AARS1*		*AD*
104	42	F	c.1574A>G	*ATL1*	Neuropathy, hereditary sensory, type ID<br># 613708	AD / AR
105	63	F	c.923C>A	*BSCL2*	Neuropathy, distal hereditary motor, type VC<br># 619112	AD / AR
106	50	F	c.1314G>C	*CAPN3*	Muscular dystrophy, limb-girdle<br># 618129	AD / AR
107	62	F	c.526G>A	*CHRNA1*	Myasthenic syndrome slowfast-channel<br># 601462	AD / AR
108	-	F	c.309C>G	*CHRNB1*	Myasthenic syndrome, congenital, 2A, slow-channel<br># 616313	AD / AR
109	6	M	c.2234A>G	*CLCN1*	Myotonia congenita, atypical, acetazolamide-responsive<br># 608390	*AD*
110	38	F	c.804+1G>A	*COL6A1*	Ullrich congenital muscular dystrophy<br>#254090	AD / AR
111	52	M	c.2966C>T	*COL6A3*	Bethlem myopathy<br># 158810	
112	40	F	c.1360C>T	DES	Myopathy, myofibrillar<br># 601419	AD / AR
113	-	M	c.822A>G<br>c.4777A>C	*DST*	Neuropathy, hereditary sensory and autonomic, type VI<br># 614653	AR
114	65	M	c.887C>T	*FLNC*	Cardiomyopathy familial hypertrophic	*AD*

					# 617047	
115	6	F	c.487C>T	*GDAP1*	Charcot-Marie-Tooth disease<br># 607831	AD / AR
116	38	F	c.677_679del			
117	49	M	c.20C>T	*HSPB1*	Charcot-Marie-Tooth disease<br># 606595	AD
118	17	M	c.244G>A	*KBTBD13*	Nemaline myopathy<br># 609273	AD
119	3	M	c.4205G>A	*MYH2*	Proximal myopathy and ophthalmoplegia<br># 605637	AD / AR
120	52	M	c.4205G>A			
121	56	M	c.3127T>G			
122	-	M	c.4421C>A	*MYH7*	Distal myopathy<br># 160500	*AD*
123	-	F	c.349G>C	*REEP1*	Neuronopathy, distal hereditary motor, type VB<br># 614751	*AD*
124	15	M	c.164C>T			
125	48	M	c.12861_12869del<br>c.12956G>A	*RYR1*	Central core disease<br># 117000	AD /AR
126	73	M	c.505T>C	*SH3TC2*	Charcot-Marie-Tooth Disease<br># 601596	AD / AR
127	44	F	c.1607G>A<br>c.1177+15T>C			
128	19	M	c.247G>T	*STIM1*	Myopathy, tubular aggregate<br># 160565	AD / AR
129	1	M	C.1568-1G>A<br>c.2147C>G	*TRAPPC11*	Muscular dystrophy, limb-girdle<br># 615356	AR
130	50	M	c.91615_91616dup	*TTN*	Muscular dystrophy, limb-girdle<br>#608807	AD / AR

Supplementary Table S4. Detailed variant description and pathogenicity classification according to ACMG guidelines.

Gen	Variant	ACMG	Very Strong	Strong				Moderate						Supporting					
			PVS1	PS1	PS2	PS3	PS4	PM1	PM2	PM3	PM4	PM5	PM6	PP1	PP2	PP3	PP4	PP5
AARS1 NM_001605.3	c.11_15del p.Thr4AsnfsTer4	**P**	■						■									
	c.986G>A p.Arg329His	**P**							■			■				■		■
ACTA1 NM_001100.3	c.580A>T p.Ile194Phe	**P**						■	■						■	■		
	c.529A>G p.Ile177Val	**P**						■	■						■	■		■
	c.809-2A>T	**P**	■						■									■
AMPD1 NM_000036.3	c.133G>T	**P**	■						■									
	c.242C>T p.Thr81Ile	**US**							■									
	c.1029A>T	**LP**							■	■						■		
ANO5 NM_213599.3	c.191dup p.Asn64LysfsTer15	**P**	■															■
	c.692G>T p.Gly231Val	**US**														■		■
	c.1119+1G>T	**P**	■						■									■
	c.206_207del p.Asp69ValfsTer9	**P**	■						■									■
	c.1982T>C p.Leu661Pro	**LP**							■							■		
	c.2235+2T>G	**P**	■						■									
	c.1767C>A p.Tyr589*	**P**	■						■									■
ATL1 NM_015915.4	c.1574A>G p.Tyr525Cys	**US**							■						■			
BSCL2 NM_001122955.3	c.923C>A p.Thr308Asn	**US**							■							■		

Gen	Variant	ACMG	Very Strong	Strong				Moderate						Supporting				
			PVS1	PS1	PS2	PS3	PS4	PM1	PM2	PM3	PM4	PM5	PM6	PP1	PP2	PP3	PP4	PP5
CAPN3 NM_000070.3	c.2361_2362insTCAT p.Arg788SerfsTer14	**P**	X						X									
	c.505C>T p.Arg169Cys	**LP**						X	X						X	X		
	c.1962delC p.Phe654fSeArg*7	**P**	X						X									X
	c.2120A>G p.Asp707Gly	**P**						X	X						X	X		X
	c.1314G>C p.Trp438Cys	**P**						X	X						X	X		
CHKB NM_005198.5	c.701C>T p.Ser234Leu	**LP**							X							X		X
CHRNA1 NM_000079.4	c.526G>A p.Val176Met	**US**							X									X
CHRNB1 NM_000747.2	c.309C>G p.Ile103Met	**US**							X									
CHRNE NM_000080.3	c.583G>C p.Asp195His	**LP**							X			X				X		
CLCN1 NM_000083.3	c.1261C>T p.Arg421Cys	**US**							X			X				X		X
	c.1214C>T p.Thr405Ile	**US**							X							X		
	c.313C>T p.(Arg105Cys)	**US**							X							X		X
	c.501C>G p.(Phe167Leu)	**LP**						X	X									X
	c.2234A>G p.Asp745Ser	**US**							X									
COL12A1 NM_004370.6	c.8215T>C p.Cys2739Arg	**LP ***							X							X		
	c.6995G>A p.Trp2332*	**P**	X						X									
COL6A1 NM_001848.2	c.868G>A p.Gly290Arg	**P**		X				X	X			X				X		X
	c.739-2A>G	**LP**	X						X									
	c.804+1G>A	**P**	X						X									X

Gen	Variant	ACMG	Very Strong	Strong				Moderate						Supporting					
			PVS1	PS1	PS2	PS3	PS4	PM1	PM2	PM3	PM4	PM5	PM6	PP1	PP2	PP3	PP4	PP5
COL6A2 NM_001849.3	c.1806C>A p.Cys602*	**P**	X						X									X
	c.2891T>C p.Met964Thr	**LP ***							X									X
	c.875G>A p.Gly292Asp	**P**						X	X			X				X		
COL6A3 NM_004369.4	c.6320_6322del p.Gly2107del	**LP**						X	X		X					X		X
	c.6210+5G>A	**P**							X		X					X		X
	c.1688A>G p.Asp563Gly	**LP**							X							X		
	c.1631G>A p.Arg544Gln	**US**							X							X		
	c.2966C>T p.Ser989Phe	**US**							X							X		
DAG1 NM_001177639	c.258G>C p.Leu86Phe	**US**							X									
**DES** NM_001927.4	c.1360C>T p.Arg454Trp	**P**				X		X	X							X		X
DMD NM_004009.3 NC_000023.10	c.7525C>T p.Gln2509Ter	**P**	X						X									X
	c.9622A>G p.Lys3208Glu	**US**							X							X		
	c.9563+1G>A	**P**	X						X									X
	c.5697dupA p.(Leu1900Ilefs*6)	**P**	X						X									X
	c.3125delA p.(Lys1042Serfs*2)	**LP**	X						X									
DNM2 NM_001005360.2	c.1856C>T p.Ser619Leu	**P**						X	X			X				X		X
DYNC1H1 NM_001376.5	c.628C>G p.His210Asp	**US**						X	X									
DPAGT1 NM_001382.4	c.1150C>G p.Leu384Val	**LP ***						X							X			
	c.574G>C p.Gly192Arg	**P**					X	X			X				X	X		X

Gen	Variant	ACMG	Very Strong	Strong				Moderate						Supporting				
			PVS1	PS1	PS2	PS3	PS4	PM1	PM2	PM3	PM4	PM5	PM6	PP1	PP2	PP3	PP4	PP5
DST NM_001723.5	c.822A>G p.Glu274Glu	**US**																
	c.4777A>C p.Ile1593Leu	**US**																
DYSF NM_003494.4	c.5429G>A p.Arg1810Lys	**P**																
FKRP NM_001039885.2	c.545A>G p.Tyr182Cys	**P**																
FLNC NM_001458.5	c.887C>T p.Ala296Val	**US**																
GDAP1 NM_018972.3	c.487C>T p.Gln163*	**P**																
	c.677_679del p.Arg226del	**P**																
GJB1 NM_000166.6	c.547C>T p.Arg183Cys	**P**																
GYG1 NM_004130.4	c.46G>C p.Ala16Pro	**LP**																
HINT1 NM_005340.6	c.110G>C p.Arg37Pro	**P**																
HSPB1 NM_001540.5	c.20C>T p.Pro7Leu	**US**																
IGHMBP2 NM_002180.2	c.181G>A p.Gly61Arg	**US**																
	c.1327C>T p.Arg443Cys	**US**																
INF2 NM_001031714.3	c.1580_1581insC p.Val530fs*49	**LP**																
KBTBD13 NM_001101362.2	c.244G>A p.Val82Met	**US**																
LAMA2 NM_000426.3	c.149C>T p.Ala50Val	**LP ***																
	c.8075+1G>A	**P**																
MFN2 NM_014874.3	c.326A>G p.Lys109Arg	**P**																

Gen	Variant	ACMG	Very Strong	Strong				Moderate						Supporting				
			PVS1	PS1	PS2	PS3	PS4	PM1	PM2	PM3	PM4	PM5	PM6	PP1	PP2	PP3	PP4	PP5
MME NM_007288.3	c.1666C>T p.Pro556Ser	**LP**							■							■		■
	c.1972G>A p.Ala658Thr	**LP**							■							■		■
MYH2 NM_017534.6	c.4205G>A p.Arg1402Gln	**US**							■							■		
	c.3127T>G p.Ser1043Ala	**US**							■									
MYH7 NM_000257.4	c.1606G>A p.Glu536Lys	**P**						■	■			■				■		
	c.409G>A p.(Glu137Lys)	**LP**						■	■							■		
	c.4421C>A p.Ala1474Asp	**US**							■							■		
PFKM NM_001166686.2	c.1820C>T p.Ser607Leu	**LP**							■							■		
PHKA1 NM_002637.4	c.2230-1G>T	**LP**	■						■									
	c.784G>A p.Val262Met	**US**							■							■		
PGAM2 NM_000290	c.365C>T p.Pro122Leu	**P**							■							■		
POMT2 NM_013382.7	c.406T>C p.Tyr136His	**US/LP**							■							■		
PYGM NM_005609.4	c.660G>A p.Gln220=	**LP**	■						■									
	c.1094C>T p.Ala365VaL	**P**							■							■		■
RAPSN NM_005055.4	c.264C>A p.Asn88Lys	**P**				■			■							■		■
REEP1 NM_022912.2	c.349G>C p.Ala117Pro	**US**							■						■	■		
	c.164C>T p.Thr55Ile	**LP**							■						■	■		

Gen	Variant	ACMG	Very Strong	Strong				Moderate						Supporting				
			PVS1	PS1	PS2	PS3	PS4	PM1	PM2	PM3	PM4	PM5	PM6	PP1	PP2	PP3	PP4	PP5
RYR1 NM_000540.2 NM_000540.3	c.10347+1G>A	**P**	■						■									■
	c.4837C>T p.Gln1613*	**P**	■						■									■
	c.7027G>A p.Gly2343Ser	**LP**							■							■		
	c.917C>T p.Ala306Val	**US**							■							■		
	c.176C>T p.Pro59Leu	**US**							■							■		
	c.6856C>G p.Leu2286Val	**US**							■							■		
	c.2709C>G p.His903Gln	**US**							■							■		
	c.12861_12869del p.Thr4288_Ala4290del	**LP**							■		■							
	c.12956G>A p.Arg4319Gln	**US**							■									
	c.7261G>T p.Ala2421Ser	**P**							■			■				■		
	c.12836C>T p.(Ala4279Val)	**US**							■									
SCN4A NM_000334.4	c.3877G>A p.Val1293Ile	**P**							■							■		■
	c.4774A>G p.Met1592Val	**P**						■	■			■				■		■
SEPN1 *(SELENON)* NM_020451	c.763C>T p.Leu255Phe	**US**							■							■		
SGCA NM_000023.4	c.409G>A p.Glu137Lys	**P**							■			■				■		■
	c.724G>T p.Val242Phe	**LP**							■			■				■		■
	c.739G>A p.Val247Met	**P**				■			■							■		■
	c.850C>T p.(Arg284Cys)	**P**							■							■		■

Gen	Variant	ACMG	Very Strong	Strong				Moderate						Supporting				
			PVS1	PS1	PS2	PS3	PS4	PM1	PM2	PM3	PM4	PM5	PM6	PP1	PP2	PP3	PP4	PP5
SGCG NM_000231.2	c.848G>A p.Cys283Tyr	**P**							■							■		■
	c.525delT p.(Phe175Leufs*20)	**P**	■						■									■
SH3TC2 NM_024577.4	c.2860C>T p.Arg954Ter	**P**	■						■									■
	c.539_541del p.Phe180del	**LP**							■		■					■		
	c.3325C>T p.Arg1109*	**P**	■						■									■
	c.2640delC p.Ala880fSer	**P**	■						■									■
	c.3511C>T p.Arg1171Cys	**P**							■							■		■
	c.1607G>A p.(Arg536Gln)	**US**							■							■		
	c.1177+15T>C	**US**														■		
	c.561_576del p.Asp188ProfsTer69	**P**	■						■									■
SLC52A2 NM_001253815.1	c.595delG p.(Ala199Hisfs*2)	**LP**	■						■									
STIM1 NM_003156.3	c.247G>T	**P**						■	■							■		
SYNE1 NM_182961.3	c.26095-1G>T	**P**	■						■									
TAZ NM_000116.5	c.527A>G p.His176Arg	**LP**							■			■			■	■		
TCAP NM_003673.3	c.255C>A p.Tyr85*	**LP**	■						■									
TPM2 NM_213674.1	c.269G>A p.Arg90His	**P**						■	■			■			■	■		■
TRAPPC11 NM_021942.5	c.1287+5G>A	**P**							■		■				■			■
	c.1568-1G>A	**P**	■												■			■
	c.2147C>G p.Ala716Gly	**US**							■									

Gen	Variant	ACMG	Very Strong	Strong				Moderate						Supporting				
			PVS1	PS1	PS2	PS3	PS4	PM1	PM2	PM3	PM4	PM5	PM6	PP1	PP2	PP3	PP4	PP5
TTN NM_001267550.2	c.38661_38665del p.12885_12887del	**P**	■						■									■
	c.3034C>T p.Arg1012*	**LP**	■						■									
	c.106531+1G>A	**P**	■						■									■
	c.33064C>T p.Arg11022*	**LP**	■						■									
	c.102941G>A p.Gly34314Asp	**LP ***							■							■		
	c.102966del p.Lys34322AsnfsTer9	**LP**	■						■									
	c.91615_91616dup p.Gly30541ProfsTer19	**P**	■						■									■
	c.38737G>T p.Glu12913*	**P**	■						■									■
	c.87019_87022del p.29007_29008del	**P**	■						■									■
	c.86992_86994delCTG insGTCTGTCAT p.Leu28998delinsValC ysHis	**LP**							■		■					■		

*Variant pathogenicity classification according PM3 (For recessive disorders, detected in trans with a pathogenic variant (Pathogenic, Moderate))

Supplementary Table S5. Global design of INMD multi-gene panel consisting of 324 genes previously associated with neuromuscular disorders: muscular dystrophies, myopathies, peripheral nerve disease, motor neuron disease, ion channel diseases and neuromuscular junction diseases.

INMD panel

AARS	ABHD12	ACADS	ACADVL	ACTA1	ADCY6	ADGRG6	ADSSL1	AEBP1	AGL	AGRN	AIFM1	ALDOA	ALG14	ALG2	ALS2	AMACR	AMPD1
ANO5	ANXA11	ARHGEF10		ASAH1	ASCC1	ATL1	ATL3	ATP2A1	ATP7A	B3GALNT2		B3GNT2	BAG3	BICD2	BIN1	BSCL2	BVES
C19ORF12		CACNA1S		CAPN3	CASQ1	CAV3	CCDC174		CCDC78	CCT5	CFL2	CHAT	CHCHD10		CHD7	CHKB	CHRNA1
CHRNB1	CHRND	CHRNE	CHRNG	CLCN1	CLN3	CNTN1	CNTNAP1		COL12A1		COL13A1	COL1A2	COL6A1	COL6A2	COL6A3	COL6A6	COLQ
COX6A1	CPT2	CRYAB	DAG1	DARS2	DCAF8	DES	DGAT2	DHTKD1	DHX30	DMD	DNA2	DNAJB2	DNAJB6	DNM2	DOK7	DPAGT1	DRP2
DST	DYNC1H1		DYSF	ECEL1	EGR2	EMD	ERBB3	ERBB4	ERGIC1	ETFA	ETFB	ETFDH	FAM111B	FBLN5	FBN2	FDX1L	FGD4
FHL1	FIG4	FKRP	FKTN	FLAD1	FLNC	GAA	GAN	GARS	GBE1	GDAP1	GFER	GFPT1	GJB1	GLDN	GLE1	GMPPB	GNB4
GNE	GOLGA2	GYG1	GYS1	GZF1	HADHA	HADHB	HARS	HINT1	HINT3	HK1	HNRNPA1		HNRNPA2B1		HNRNPDL		HOXD10
HRAS	HSPB1	HSPB3	HSPB8	IBA57	IGHMBP2		IKBKAP	INF2	INPP5K	ISCU	ISPD	ITGA7	JPH1	KARS	KBTBD13	KIF1A	KIF1B
KIF5A	KLC2	KLHL40	KLHL41	KY	LAMA2	LAMB2	LAMP2	LARGE	LAS1L	LDB3	LDHA	LGI4	LIMS2	LITAF	LMNA	LMOD3	LRP4
LRSAM1	LTC4S	MARS	MATR3	MCM3AP		MED25	MEGF10	MFN2	MICU1	MME	MORC2	MPZ	MSTO1	MTM1	MTMR14	MTMR2	MUSK
MYBPC1	MYF6	MYH14	MYH2	MYH3	MYH7	MYH8	MYO18B	MYO9A	MYOT	MYPN	NAGLU	NALCN	NAPB	NDRG1	NEB	NEFH	NEFL
NEK1	NEK9	NGF	NTRK1	OPA1	ORAI1	PABPN1	PDK3	PFKM	PGAM2	PGK1	PHKA1	PHKB	PIEZO2	PIP5K1C	PLEC	PLEKHG5	
PMP22	PNPLA2	PNPLA6	PNPLA8	POGLUT1		POLG	POMGNT1		POMGNT2		POMK	POMT1	POMT2	PRPS1	PRX	PTPLA	PUS1
PYGM	PYROXD1		RAB7A	RAPSN	RBCK1	REEP1	RETREG1		RYR1	SBF1	SBF2	SCN10A	SCN4A	SCP2	SEPN1	SETX	SGCA
SGCB	SGCD	SGCG	SH3TC2	SIGMAR1		SLC12A6	SLC18A3	SLC22A5	SLC25A3	SLC25A32		SLC25A42		SLC25A46		SLC3A1	SLC52A1
SLC52A2	SLC52A3	SLC5A7	SMCHD1	SNAP25	SOD1	SPEG	SPG11	SPTBN4	SPTLC1	SPTLC2	SQSTM1	STAC3	STIM1	SURF1	SYNE1	SYNE2	SYT2
TARDBP	TAZ	TBCD	TBCE	TBCK	TCAP	TDP1	TFG	TIA1	TK2	TMEM43	TMEM5	TNNI2	TNNT1	TNNT3	TNPO3	TNXB	TOR1AIP1
TOR1AIP2		TPM2	TPM3	TRAPPC11		TRIM2	TRIM32	TRIM54	TRIM63	TRIP4	TRPV4	TTN	TTR	UBA1	UNC80	VCP	VMA21
VRK1	WARS	WNK1	YARS	YARS2	ZAK	ZBTB42											
